# Predictors of two forms of attrition in a longitudinal health study involving ageing participants: An analysis based on the Whitehall II study

**DOI:** 10.1186/1471-2288-12-164

**Published:** 2012-10-29

**Authors:** Gill Mein, Suneeta Johal, Robert L Grant, Clive Seale, Richard Ashcroft, Anthea Tinker

**Affiliations:** 1 Faculty of Health and Social Care Sciences, St. George's University of London and Kingston University, London, England, SW17 0RE, UK; 2 Department of Sociology and Communications, Brunel University, Uxbridge Middlesex, England, UB8 3PH, UK; 3 School of Law, Queen Mary University of London, London, England, UK; 4 Institute of Gerontology, King's College London, London, England, UK

**Keywords:** Older people, Attrition, Longitudinal studies, Retention, Whitehall II study

## Abstract

**Background:**

Longitudinal studies are crucial providers of information about the needs of an ageing population, but their external validity is affected if partipants drop out. Previous research has identified older age, impaired cognitive function, lower educational level, living alone, fewer social activities, and lower socio-economic status as predictors of attrition.

**Methods:**

This project examined attrition in participants of the Whitehall II study aged between 51–71 years, using data from questionnaires participants have completed biennially since 1985 when the study began. We examine the possibility of two distinct forms of attrition – non-response and formally requesting to withdraw – and whether they have different predictors. Potential predictors were age, gender, marital status, occupational grade, retirement, home ownership, presence of longstanding illness, SF-36 quality of life scores, social participation and educational level comparing participants and those who had withdrawn from the study.

**Results:**

The two forms of attrition share many predictors and are associated but remain distinct. Being older, male, having a lower job grade, not being a home owner, not having a long standing illness, having higher levels of education, and not having retired, were all associated with a greater probability of non-response; being married was associated with higher probability in women and lower in men. Being older, male, having a lower job grade, not being a home owner, having lower SF-36 scores, taking part in fewer social activities, and not having a long standing illness, were all associated with greater probability of withdrawal.

**Conclusions:**

The results suggest a strong gender effect on both routes not previously considered in analyses of attrition. Investigators of longitudinal studies should take measures to retain older participants and lower level socio-economic participants, who are more likely to cease participating. Recognition should be given to the tendency for people with health problems to be more diligent participants in studies with a medical screening aspect, and for those with lower socio-economic status (including home ownership), quality of life and social participation, to be more likely to request withdrawal. Without taking these features into account, bias and loss of power could affect statistical analyses.

## Background

Longitudinal studies are important in gaining an understanding of the ageing process in older people. This is particularly important as life expectancy and the proportion of older people in the population continues to increase [1-4]. However, attrition in such studies can create bias in their samples and affect the validity of the study if those who drop out differ in characteristics from remaining participants [5-7].

The authors previously reviewed the literature on attrition in longitudinal studies and found that those who drop out from studies tend to differ from those who continue to participate [8]. Studies have demonstrated that attrition was associated with being older and being cognitively impaired, with having poor cognitive functioning, living alone and not being married, lower socio-economic status or level of education and being less socially active [8,9]. There was little or no clear evidence about the relationship between attrition and factors such as gender, health and home ownership [8,9].

In this paper we report on our analysis of attrition in data collected by the Whitehall II Study. The Whitehall II study is a longitudinal research study, which began in 1985 and recruited 10,308 participants aged between 35–55 years from 20 London based civil service departments. Since then, participants have self-completed questionnaires on health, work and lifestyle every two years and undergone a medical examination every five years [10]. These data collections are known as “phases”. Continued participation in the Whitehall II study is good, although as participants age it is expected to reduce.

We sought to answer the research question: what factors significantly predict attrition from Whitehall II and what is the most predictive model that can be formed from them? We divided the general concept of attrition (dropout) into two forms, non-response and formally requesting to withdraw. The authors are not aware of any previous research that made this distinction, despite a broad systematic review of the literature. Because previous research has indicated that grouping mortality and non-mortality related dropout together may create a highly selective picture of attrition, we did not count participants who had died in either of these categories [11,12].

## Methods

### Source of data

Ethical approval for the Whitehall II study was granted by the University College London Medical School committee on the ethics of human research.

Data in this analysis was from participants who were then aged 51–71 years, with the usual age of 60 years for retirement from the civil service. Of the 10,308 participants at baseline, a total of 1,377 (13%) had formally requested to be withdrawn from the study by phase 6. Non-response was defined at each phase as a binary outcome, and individuals could return to participation after a period of non-response. Table 1 shows the combined impact of this formal withdrawal, death and simple non-response at each phase.

**Table 1:** **Phases of the Whitehall II cohort study (**http://www.ucl.ac.uk/whitehallII/study-phases**)**

**Phase**	**Dates**	**Type**	**Number of participants**	**Response Rate**
1	1985-1988	Screening / questionnaire	10,308	73% of those invited
2	1989-1990	Questionnaire	8,132	79% of Phase 1 responders
3	1991-1994	Screening / questionnaire	8,815	86% of Phase 1 responders
4	1995-1996	Questionnaire	8,628	84% of Phase 1 responders
5	1997-1999	Screening / questionnaire	7,870	76% of Phase 1 responders
6	2001	Questionnaire	7,355	71% of Phase 1 responders
7	2002-2004	Screening / questionnaire	6,967	68% of Phase 1 responders
8	2006	Questionnaire	7,173	70% of Phase 1 responders
9	2007-2009	Screening / questionnaire	6,761	66% of Phase 1 responders

The precise date of withdrawal was known for 563 of 1377 who formally requested to withdraw from the cohort. The distribution of these dates was quite different from the dates of last response, and in particular rarely appeared in phases 1–3. The dates of last response were also notably different for those who withdrew and had a date recorded, compared to those who withdrew without a date. Tracing of non-responders became more systematic and sophisticated at phase 4. This suggests that the distribution of recorded withdrawal dates overlaps only very slightly with that of the unrecorded ones, making imputation inappropriate. We therefore treated the missing withdrawal dates as left-censored (known to have occurred at some point up to Phase 4), and the analysis of withdrawal started for all participants at the end of phase 4.

### Predictor variables

The choice of variables was guided by our literature review [8,9]. All of the information comprising these variables was obtained from questionnaires completed by the participants. Gender, marital status, home ownership, occupational grade, longstanding illness and age were all measured at the baseline measurement phase 1 (1985–1988) except for educational level - where a better measure of this was used at phase 5, and social participation - where data had only been collected at phase 5 (1997–1999). The other variables were collected at each phase of the study (Table 2).

**Table 2:** Potential predictors of attrition variables

**Potential predictors of attrition variables**	**When obtained from self-completed questionnaires**	**Response rate**
Age (years)	Phase 1 (1985–1988)	10,308
Gender	Phase 1 ( 1985-1988	10,308
Marital status (Married/not married)	Phase 1 ( 1985-1988	10,308
Occupational grade (3 levels)	Phase 1 ( 1985-1988	10,308
Educational achievement (5 levels)	Phase 5 (1997–1999)	7,870
Retirement	At various phases	
Housing (owned/rented)	Phase 1 ( 1985-1988	10,308
Number of social activities	Phase 5 (1997–1999)	7,870
SF 36 (mental health & physical function)	Phase 5 (1997–1999)	7,870
Longstanding illness (self-reported yes/no)	Phase 1 ( 1985–1988) only asked of 6339 participants	10,308

Educational level was measured on a 5 point scale ranging from having no educational qualifications to having a university degree. Occupational grade ranged from low (clerical and support grades) to high (senior administrative grades). The presence of longstanding illness was subjectively reported by participants. This question was only asked in later versions of the phase 1 questionnaire, and therefore was answered by 6339 people at Phase 1.

### Imputation and assumptions

The extent of missing data within phases where a participant responded is small but perhaps not negligible, especially as we will use the variables described above as explanatory variables in regression analyses. There is a general problem in imputing missing data where non-response or withdrawal is the outcome of interest. Because we believe all the explanatory variables could affect “missingness”, the data are therefore “missing not at random” (MNAR) by Rubin’s taxonomy [13], which means that it is not possible to use the data we do know to fill in plausible values for those that are missing, because those that are missing are liable to be different in some way that our data cannot predict. A further complexity arises from the longitudinal nature of the data, which needs to be taken into account when imputing. Multiple imputation, or the weighted estimating equation approach [14], was also not used in these analyses because none of the covariates were missing in a proportion of participants so large as to cause concern about bias.

Year of birth is calculated from age in any given phase and date of response, with the exception of 15 participants who had no date of response, and for these we assumed the median date of the phase. Whitehall II did not collect a precise date of retirement; we could detect an approximate time from the first phase when a participant said they were retired, but this question was only introduced from phase 4 onwards. Where this date cannot be approximated, either because the participant was non-responding from phase 4 on, or because they had already retired at phase 4, it was imputed using the median age at the first phase after retirement (60.8 years). This is a single imputation and liable to underestimate the uncertainty in any effect of retirement on non-response / withdrawal, but because all data are observed only in phases, the exact date of retirement does not matter as long as it is not allocated to completely the wrong phase of the study, and we believe this is unlikely. Table 3a shows the impact of missing data on key time-varying covariates.

**Table 3:** Numbers of participants with complete data for various time-varying covariates

**Phase**	**N participating**	**N not retired**	**Employment grade recorded**	**Marital status recorded**	**Housing tenure recorded**	**SF36 recorded**	**Long-standing illness recorded**
1	10308	N/A	10308	10270	10226	N/A	7654
2	8132	N/A	8110	8124	8095	N/A	8129
3	8815	N/A	8312	8306	8307	8292	8634
4	8628	7021	5386	7801	N/A	7669	8564
5	7870	5625	3525	6921	N/A	6927	7250
6	7355	4543	2595	6665	N/A	6601	7345

The number of social/cultural activities summarises a group of questions asked at phase 5 only, and recorded for 7142/7870 participants. By counting them, we assume they are of equal (and additive) importance in predicting non-response and time to withdrawal. We also assume that phase 5 provides a measure that suitably represents the participants throughout the study.

Employment grade, marital status and home ownership were imputed by last observation carried forward where not recorded. Long-term illness was imputed by carrying forward and backwards and analyses repeated; the method was not found to affect results substantively, so backward imputation was used for transient non-response because illness is a plausible cause.

### Statistical analyses

Non-response is a longitudinal binary outcome and was modelled as such over phases 1–6 using a multilevel logistic regression model with the individual participant as the higher level under which are the phases of the study. This allowed us to estimate effects of the predictors, unbiased by the auto-correlation in the longitudinal data. The outcome is missing for phases after the participant has died or withdrawn, because they are no longer at “risk” of non-response. The explanatory variables were added in the order: gender, age, age squared, occupational grade, education, retirement, marital status, house ownership, social activities, SF-36 (both sub-scales included or removed from the model together as a block), and long-standing illness. Interactions were considered between sex and each of the following: employment grade, education, marital status, and retirement as soon as both variables were included. The “gllamm” program for Stata software was used to fit the model and predictions for individuals from the random effect were extracted using the mean best linear unbiased predictor (BLUP), derived from the “gllapred” program for Stata [15]. This gives an empirical Bayes estimate of each individual’s log-odds ratio for non-response compared to the average for the cohort, after independent variables have been accounted for [16].

Withdrawal is a terminal event and there are participants who do not have a withdrawal date either through remaining in the study or death. Because withdrawal dates are clustered around the data collection periods for each phase, a Cox proportional hazards model of time to withdrawal was appropriate and was fitted using Stata version 11 software. The period of time under consideration was from the participant’s response to phase 4 (or the median date of phase 4: 18 May 1995, if they did not respond) to the earliest of withdrawal, death or 1 January 2009. Hazard of withdrawal will differ as a result of administrative activities, correspondence, tracing exercises and data collection phases, so the calendar date was used as the timescale. Predictor variables were added in the same order as for the logistic regression, and the participants’ BLUPs were added to the final model as an additional predictor. Time-varying covariates were as described above under “Predictor Variables”.

## Results

The model for non-response could be fitted on 9042 participants who had complete data on the predictor variables. Because of the large number of data and complexity of the computation required, this took 7.5 hours to converge to a solution on a 2.13GHz Intel Core2 desktop computer. The model was fitted using 3, 6 and 30 integration points to test stability of the results [15]; only negligible differences were seen, and the results in Table 4 are for 30 integration points. Predictors and interactions not shown in the table were not significant, along with the quadratic effect of age.

**Table 4:** Results of the longitudinal analysis of non-response

			**Odds Ratio**	**p-value**	**95% CI**
Age			1.08	<0.001	1.07-1.09
Sex	Male	(baseline)			
	Female		0.60	<0.001	0.48-0.75
Educational level	Primary	(baseline)			
	Secondary		1.23	0.007	1.06-1.44
	Tertiary		1.67	<0.001	1.41-1.96
Employment grade	High	(baseline)			
	Middle		2.36	<0.001	2.04-2.72
	Low		5.43	<0.001	4.45-6.63
Retired	(in men)		0.48	<0.001	0.41-0.57
	(in women)		0.78	0.007	0.66-0.93
Married	(in men)		0.74	<0.001	0.63-0.87
	(in women)		1.25	0.019	1.04-1.51
Homeowner			0.54	<0.001	0.45-0.65
Long-standing illness			0.68	<0.001	0.62-0.74

The random effect models inter-participant variability (in terms of the logarithm of the odds of non-response) by a normal distribution, which has a standard deviation of 2.01. Because 95% of data in a normal distribution lie within 1.96 standard deviations of the mean, as many participants have odds of non-response between 51 times greater and 51 times smaller than the predicted value (e^1.96x2.01^=51), with residual intra-class correlation of 0.65 (95% CI 0.63-0.66). This indicates a very large inter-participant variability, which could be interpreted as a propensity to respond/ not respond. Each participant’s propensity to non-response was predicted from the random effect using the BLUP.

We included 9259 participants in the Cox regression model for time to withdrawal, with 520 withdrawals, making a total of 118,944 person-years at risk. The proportional hazards assumption was tested graphically and found to be acceptable (Table 5).

**Table 5:** Results of the survival analysis of withdrawal

			**Hazard Ratio**	**p-value**	**95% CI**
Age			1.08	<0.001	1.05-1.11
Sex	Male	(baseline)			
	Female		0.63	0.029	0.41-0.95
Employment grade	High	(baseline)			
	Middle		1.31	0.196	0.87-1.99
	Low		1.91	0.026	1.08-3.39
Homeowner			0.61	0.068	0.36-1.04
Social activities			0.88	0.002	0.81-0.95
SF-36 physical			0.98	0.023	0.96-1.00
SF-36 mental			0.97	<0.001	0.95-0.99
Long-standing illness			0.68	0.045	0.47-0.99

The odds ratios and hazard ratios cannot be quantitatively compared as they measure different things. However, it is clear that the qualitative effects of age, sex, employment grade, home ownership and long-standing illness are shared between both models. Both forms of attrition are more likely in participants who are older, male, in lower employment grades, do not own their own home, and free from long-standing illness. Educational level, retirement and marital status appear to affect non-response but not withdrawal. In contrast, social activities and SF-36 scores for physical function and mental health were associated with withdrawal but not non-response.

**Figure 1:**
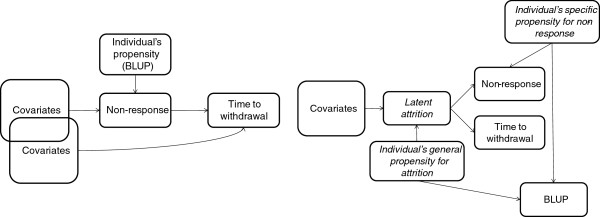
Two plausible causal models linking non-response and withdrawal.

We also investigated the relationship between the two forms of attrition to some extent by including the BLUP (individual propensity to non-response) in the survival model for withdrawal. This is a significant predictor in addition to those in Table 5, and its inclusion does not materially alter any of the other coefficients. This suggests that the BLUPs predict withdrawal as well as non-response and are not on the causal pathway from predictors to withdrawal (via non-response) [17]. There are two alternative interpretations of the relationship, shown in Figure 1. Either non-response directly causes withdrawal, at least in part, or they share an unobserved latent variable representing general attrition. The latter could involve a general and a specific form of individual propensity, but with the general form unobserved, both will be combined to some extent in the BLUP, and so it is through the general propensity that the BLUP would predict withdrawal. In practical terms they are indistinguishable because the latent variable is unobservable, but the differences in covariates between the two outcomes, as well as the temporal order of the outcomes, argue against the “latent attrition” model, which would have a single set of individuals’ characteristics affecting both outcomes.

## Discussion

The purpose of this study was to examine whether the characteristics of participants and dropouts in the Whitehall II study differ. We have found a large effect of gender, not established in previous analyses of attrition. Men are considerably more likely to drop out, all other factors being equal. There is an interesting effect of retirement and long-standing illness, both of which appear to reduce non-response, with long-standing illness also reducing withdrawal. The effect of educational level on non-response is unexpected, with higher qualifications associated with more non-response. This could be attributable to greater mobility in this group, particularly around retirement, causing the study to lose contact with them, but this effect needs to be examined further to be properly understood.

The effect of lower SF-36 scores and fewer social activities appears to increase withdrawal – but not non-response – which confirms and adds detail to previous research where cognitive impairment and social isolation were associated with dropout. These results add some additional detail in suggesting that self-reported long-standing illness, a health concern that is not necessarily debilitating or socially isolating, actually reduces both forms of attrition while reduced physical function, mental health and social activity increases withdrawal from the study. It would be useful to discover more detail about the long-standing illnesses in the cohort.

An analytic method gaining popularity is joint modelling of longitudinal and time-to-event processes [18]. While this is an interesting potential avenue for further investigation of these data, there are three major methodological barriers to be overcome before it could be employed. Firstly, at the time of writing, such models have yet to be demonstrated on autocorrelated binary longitudinal processes. Though the extension from continuous variables such as biomarkers is theoretically straightforward, the implementation in software requires considerable work. Secondly, the time to withdrawal is subject to some assumptions as set out above. While we believe the assumptions provide results sufficiently approximating the underlying processes, in a joint model it would be worth including the uncertainty about withdrawal as an interval-censored time variable, and this has not been done before. Thirdly, there are no software packages currently available that allow joint modelling beyond the basic specification, and so each model has to be written as a bespoke program. We believe the reasonable approximation we have achieved does not warrant additional analyses on this scale.

These findings support those from other longitudinal studies involving older people, reviewed in the introduction. Findings from research into dropout in other longitudinal studies have not been consistent on the relationship of gender, health status or home ownership with attrition. Our results add considerable detail to the evidence base on this topic.

### Implications of results

Attrition is a major issue in research, particularly in longitudinal studies involving older people, where each phase is likely to involve further dropout and the possibility of sample bias if those who drop out differ in characteristics from remaining participants. These findings have implications for those who plan longitudinal studies, who must be prepared to expect higher attrition or refusal rates from these groups of people. One way of tackling this is oversampling from within these groups at the initial phase of recruitment, and appropriately weighting the data in any analyses. This would ensure that when people from these groups are lost at follow up, there are still adequate numbers who remain as participants. Oversampling of these “at risk” groups at baseline would be difficult as the variables are not known until interview. Encouragement to continue participating is paramount especially as participants age and their participation becomes more difficult. Methods for doing this (to ensure the sample remains representative) - particularly for older people - are discussed elsewhere [8]. It is especially important to encourage older people to continue participating in longitudinal studies if we are to use research findings to understand the needs of this group. As life expectancy and the proportion of older people in the population continues to increase, it is vital to gain an understanding of the ageing process to inform policy decisions regarding current and future generations of older people. However, methods for dealing with missing data to achieve unbiased statistical results, such as multiple imputation or doubly robust estimators, are increasingly widely used, and these rely on models that effectively predict missing data. Our findings provide an indication of the variables to collect in order to construct such models, as well as evidence that different models should be considered in longitudinal studies where two types of attrition may occur.

### Strengths and limitations

A strength of this study is the large sample size used for the analysis. Other analyses of attrition in longitudinal studies involving older people have been based on much smaller samples [7,9]. In addition, the study population is homogenous with respect to occupation, yet the civil service grading structure provides a clear ranking by socio-economic standing. However, restriction of the sample to members of the civil service excludes certain categories of people such as the unemployed. Original recruitment for the Whitehall study was from Central London based departments, which perhaps restricts its generalisability to the rest of the population.

Our study was limited by the variables available in the Whitehall II dataset and the completeness of these data. For example, we wished to examine the relationship between cognitive function and attrition in the study, but the cognitive function measures were only introduced in later phases of the study, after a substantial number of participants had already requested to be withdrawn. Also, participants had not been systematically asked for reasons for withdrawal or when returning after a period of non-response.

### Future research

Further research is required to address the issue of attrition, and to find ways of encouraging retention. In particular, research on the effectiveness of different ways of encouraging people of low socio-economic standing to participate in longitudinal studies, and to stay within these studies, is needed. Researchers involved in longitudinal studies should record the extent of attrition in their studies, and details of those who cease to participate and their reasons for doing so. This would ensure that further evidence on the reasons for attrition becomes available. The distinction between predictors of non-response and withdrawal needs to be confirmed in other longitudinal studies. Development of methods for joint modelling may also shed light on the relationship in the near future.

## Conclusions

In conclusion, attrition in longitudinal studies is a serious issue as samples become biased and affect the validity of the study, if those who drop out differ from participants. We found that those who dropped out of the Whitehall II study did differ in characteristics from those who continued to participate. In particular, after controlling for all other variables, we found that those who were older and from a low occupational grade were more likely to drop out.

## Abbreviations

MNAR: Missing not at random; BLUP: Best linear unbiased predictor.

## Competing interests

The Principal Investigator Anthea Tinker is a participant in the Whitehall II study. This was declared to the appropriate ethics committee. All of the data was anonymised.

## Authors' contributions

GM conceived the original study and revised the final manuscript, RG conducted the final statistical analysis, SJ did the initial analysis and wrote the first draft, CS did the initial analysis and wrote the first draft. RA read all versions of the manuscript, AT conceived the original study, read and edited all versions of the manuscript. All authors have read and approved the final manuscript.

## Declaration of sources of funding

This work was supported by The Atlantic Philanthropies, [grant number 15867] and we express our gratitude to them.

The Whitehall II study has been supported by grants from the Medical Research Council; British Heart Foundation; Health and Safety Executive; Department of Health; National Heart Lung and Blood Institute (HL36310), U.S. National Institutes of Health: National Institute on Aging [AG13196], U.S. National Institutes of Health; Agency for Health Care Policy Research [HS06516]; and the John D and Catherine T MacArthur Foundation Research Networks on Successful Midlife Development and Socio-economic Status and Health. We thank all participating Civil Service departments and their welfare, personnel, and establishment officers; the Occupational Health and Safety Agency; the Council of Civil Service Unions; all participating civil servants in the Whitehall II study; all members of the Whitehall II study team.

## Pre-publication history

The pre-publication history for this paper can be accessed here:

http://www.biomedcentral.com/1471-2288/12/164/prepub
